# Inactivation of Foodborne Viruses by UV Light: A Review

**DOI:** 10.3390/foods10123141

**Published:** 2021-12-18

**Authors:** Vicente M. Gómez-López, Eric Jubinville, María Isabel Rodríguez-López, Mathilde Trudel-Ferland, Simon Bouchard, Julie Jean

**Affiliations:** 1 Catedra Alimentos para la Salud, Universidad Católica San Antonio de Murcia, Campus de los Jerónimos, E-30107 Murcia, Spain; vmgomez@ucam.edu; 2 Institute of Nutraceuticals and Functional Foods, Département des Sciences des Aliments, Université Laval, Québec, QC G1V 0A6, Canada; eric.jubinville.1@ulaval.ca (E.J.); mathilde.trudel-ferland.1@ulaval.ca (M.T.-F.); simon.bouchard.13@ulaval.ca (S.B.); 3 Departamento de Tecnología de la Alimentación y Nutrición, Universidad Católica San Antonio de Murcia, Campus de los Jerónimos, E-30107 Murcia, Spain; mirodriguez@ucam.edu

**Keywords:** UV light, pulsed light, foodborne viruses, mechanism of inactivation, food safety

## Abstract

Viruses on some foods can be inactivated by exposure to ultraviolet (UV) light. This green technology has little impact on product quality and, thus, could be used to increase food safety. While its bactericidal effect has been studied extensively, little is known about the viricidal effect of UV on foods. The mechanism of viral inactivation by UV results mainly from an alteration of the genetic material (DNA or RNA) within the viral capsid and, to a lesser extent, by modifying major and minor viral proteins of the capsid. In this review, we examine the potential of UV treatment as a means of inactivating viruses on food processing surfaces and different foods. The most common foodborne viruses and their laboratory surrogates; further explanation on the inactivation mechanism and its efficacy in water, liquid foods, meat products, fruits, and vegetables; and the prospects for the commercial application of this technology are discussed. Lastly, we describe UV’s limitations and legislation surrounding its use. Based on our review of the literature, viral inactivation in water seems to be particularly effective. While consistent inactivation through turbid liquid food or the entire surface of irregular food matrices is more challenging, some treatments on different food matrices seem promising.

## 1. Introduction

Ultraviolet (UV) light refers to the portion of the electromagnetic spectrum that falls in the wavelength range of 100–400 nm. This band is divided into vacuum UV (100–200 nm), UV-C (200–280 nm), UV-B (280–315 nm) and UV-A (315–400 nm). The range useful for inactivating viruses and microorganisms includes UV-C and UV-B up to about 300 nm. The 100–200 nm sub-band can render viruses non-infectious, but only in a vacuum, since these wavelengths are readily absorbed by air.

Artificial UV light can be provided by lamps. The most common type is the low-pressure mercury lamp, which emits a quasi-monochromatic output at 253.7 nm. Medium-pressure mercury lamps with polychromatic emission, light-emitting diodes that generate narrow bandwidth emissions in different ranges and excimer lamps containing a rare gas halide such as KrCl* (222 nm) or XeBr* (282 nm) are also available [1].

A special case of light-based disinfecting technology is pulsed light. These systems generate flashes of high-intensity polychromatic light ranging from UV to infrared by storing electric energy in capacitors, which are discharged to a xenon lamp [2]. Pulsed light can inactivate viruses faster than conventional light sources and is generally more efficient than low or medium pressure lamps on a time and fluence basis [3,4].

Viral inactivation by UV light is a function of the energy impinging on the targeted surface, called fluence (*F*, J/cm^2^), defined formally as “the total radiant energy traversing a small transparent imaginary spherical target containing the point under consideration, divided by the cross section of this target” [5]. If the fluence rate (*E*) is constant over time, the fluence is determined according to the following equation:

F=Et

where *t* is the exposure time in seconds. For the specific case of pulsed light technology,

$$F=F_{p}n$$

where *F_p_* is the fluence per pulse and *n* the number of pulses [6].

The use of UV light for water disinfection, including destruction of viruses, is a well-established industrial practice that has been in continuous use for more than 50 years [7] to process as much as 8.5 billion gallons of potable water per day [8]. The use of UV light to inactivate bacteria in foods has been studied extensively [9,10], while its potential for inactivating viruses in or on foods has received less attention.

According to the latest World Health Organization (WHO) report on the subject, foodborne diseases are a significant global burden causing high rates of morbidity and mortality [11]. At least 31 foodborne agents, mainly microbial, often viral and sometimes chemical, caused an estimated 600 million illnesses in 2010 [11]. In addition to disastrous consequences for public health, contamination of food by viruses remains a significant economic issue for stakeholders in the food industry, where recall costs and drops in sales can be financially ruinous [12]. In the United States and Canada, more than 20 recalls due to foodborne viruses were issued from 2017 to 2021, specifically for frozen berries and shellfish [13,14,15]. Despite the prevalence of foodborne viral illness, interest in this domain has emerged relatively recently. It was not until 2008 that the Food and Agriculture Organization (FAO) in collaboration with the WHO concluded that viruses were causing an increasing proportion of foodborne infectious intestinal diseases. The scarcity of epidemiological data, the difficulty of detection, persistence on food contact surfaces and limited post-harvest decontamination options were all identified as contributors to the emerging problem. Two viruses stood out for their number of cases and the severity of their symptoms: human noroviruses (HuNoV) and hepatitis A virus (HAV) [16]. It is estimated that the number of foodborne illnesses caused by HuNoV annually has passed 124 million cases with 34,000 deaths worldwide [11]. HuNoV appears to be causing 58% of foodborne illnesses in the USA [17] and 64% in Europe and the British Isles [18]. For HAV, the number of foodborne cases is smaller (14 million worldwide), but the mortality rate is 10 times higher (28,000 per year) and its incidence is increasing at a worrying pace in and around Europe [11,19]. HAV is transmitted via various routes starting from feces, including contaminated irrigation water, fishing sites or infected food handlers (symptomatic or not) [20,21,22]. Ready-to-eat and minimally processed foods such as shellfish, vegetables, and fruits are often implicated in HuNoV and HAV outbreaks [16]. During the past decade, berries, particularly frozen, have been at the origin of several major outbreaks of both viruses around the world [23,24], including one of the largest outbreaks ever of HuNoV (>11,000 cases), which occurred in Germany in 2012, believed to be caused by strawberries from China [25]. In 2013, frozen berries were the source of a large and prolonged multistate European outbreak of HAV centralized in Italy with more than 1800 cases [26,27]. Green onions were the source of an outbreak of HAV that led to 601 cases and 3 deaths in the United States in 2003 [28]. The mollusc origin of several epidemics around the world has shown the significance of contaminated water as a vector of HuNoV [29,30,31] and HAV [32,33]. Involvement of food handlers served in public is also reported frequently [19,34]. In recent years, public health authorities have noted an increase in cases of hepatitis E virus (HEV), an emerging foodborne virus, particularly in countries with high standards of hygiene [35,36]. Compared to HuNoV and HAV, outbreaks of HEV tend to be small and isolated [19]. HEV has been found in seafood [37,38,39,40], fresh and frozen produce [40,41,42] and, more recently, in the milk of different animals [43,44]. However, pork and game such as wild boar and deer are the sources most often implicated in foodborne cases [19,45]. Although HEV is the most common hepatitis virus associated with meat products, HAV has been detected in processed and raw meats in South America [46] and 117 cases of HuNoV were traced to an infected handler employed in the production of a ready-to-eat meat product [47].

In this review, we examine inactivation of the most important foodborne viruses and their laboratory surrogates by UV light and discuss the commercial applicability of this technology to specific food types and food contact surfaces.

## 2. Definition of Viruses

Viruses are lifeforms that have their genome (single-stranded or double-stranded DNA or RNA) coiled with a protein into a structure called a nucleocapsid, which may be enveloped in a lipid membrane [48]. They are classified phylogenetically in orders, families and genera defined by their most salient taxonomic characteristics [49]. Most virions (single infectious viral particles) have an icosahedral or helical structure [50] with or without external features such as spike proteins [51]. Some have a segmented genome [52]. The length of the genome ranges from 1682 nucleotides (Hepatitis delta virus) to 2,473,870 nucleotides (*Pandoravirus salinus*) [53]. The average length of foodborne virus genomes is about 7500 nucleotides. Virions range in size from about 17 nm to 1.5 μm in average diameter [54]. Viruses are obligate intracellular parasites, their life cycle consisting of infecting cells of the host, replicating therein, emerging therefrom and infecting other cells or being transmitted to a new host [55]. Nearly all living organisms, including mammals, birds, reptiles, amphibians, fish, bacteria, archaea, and plants, can be infected by viruses. Many viruses can be propagated in suitably adapted cell cultures [56,57].

Each virus infects a limited range of host species and tissue or cell types. The surface structures of a virus allow it to attach to specific cell receptors composed of proteins, carbohydrates and/or lipids [58]. For example, norovirus (NoV) has two surface proteins, VP1 and VP2, that recognize cells in the human digestive tract [59]. After attachment, the viral genome is released from the capsid into the cell by direct fusion or receptor-mediated endocytosis. The invading DNA or RNA then commandeers the cell cytoplasm or nucleus to direct the synthesis of genome replicates and new viral proteins using host cell ribosomes [60]. The assembled new virions then emerge from the infected cell, in some cases causing cell lysis [61].

Viruses in general and particularly foodborne viruses are tenacious because of their ability to persist in the environment, their adhesive properties and the low infectious titer required for pathogenicity. Some viruses remain infectious over a wide range of pH, temperature and humidity and tolerate freezing and various physical and chemical treatments [62]. Non-enveloped viruses (e.g., foodborne) are more resistant than enveloped types [63], the lipid membrane being less stable than the capsid. Detergents are very effective at preventing infection by most enveloped viruses [63]. Adhesion to surfaces is a major facilitator of viral propagation. This factor depends mainly on electrostatic and hydrophobic forces, which are influenced by environmental conditions such as ionic strength, pH, and the presence or absence of surfactant substances [64]. Over the past decade, viruses have become a major food safety concern.

## 3. The Most Relevant Foodborne Viruses

### 3.1. Human Noroviruses

Human noroviruses belong to the *Caliciviridae* family, which are non-enveloped viruses with a positive-stranded RNA genome of approximately 7.5–7.7 kilobases [65]. Their impressive genetic diversity comprises more than 10 genogroups (GI to GX) currently represented by 48 genotypes [66]. Only a few specific genotypes, namely GI, GII, GIV, GVIII and GIX, are known to affect humans [67]. The only variant known to be prevalent worldwide is GII.4 [68,69], although recent data show that GII.17 has become an emergent dominant strain [70]. In China, GII has been found to be nine times more prevalent than GI [71]. Much work has been devoted to developing an immunization tool. However, no HuNoV vaccine is currently beyond phase 2 clinical trials [72,73,74,75]. Vaccine developers face the challenge of a high mutation rate [76]. In addition, since the infectious dose of HuNoV is 10–100 virions [77], controlling the spread of this virus will likely continue to rely heavily on inactivation strategies applied in the environment.

### 3.2. Hepatitis A Virus

HAV are quasi-enveloped viruses belonging to the *Picornaviridae* family [78]. They have a single-stranded positive-sense genome approximately 7.5 kb in length [79,80,81]. Unlike HuNoV, the number of genotypes is small (6), and only three affect humans (I, II, and III). These are divided into subtypes A and B [82]. Genotype I is the most prevalent [36]. With only one circulating serotype [83], the development of an effective vaccine was relatively easy [84,85,86]. Distinguishable strains circulate variably in different countries [87]. HAV epidemiology appears to differ considerably between industrialized and developing countries [88]. Due to vaccination, HAV circulates much less in developed countries. However, the younger population may have never been exposed to HAV or vaccinated, making them potentially susceptible to infection [36]. Since most outbreaks of HAV in developed countries originate from developing countries [89], due mainly to increased importation of produce [90], improvements in global sanitary standards and viral control methods are needed to limit the re-emergence of HAV where it is not endemic.

### 3.3. Hepatitis E Virus

An emerging cause of foodborne hepatitis is the E virus [36]. HEV is a quasi-enveloped virus belonging to the *Hepatoviridae* family [91]. It is a positive-stranded RNA virus with a genome size of 7.2 kb [91]. Eight genotypes are known. Only HEV1 and HEV2 are obligate human pathogens, while HEV3 and HEV4 are zoonotic [91]. An increasing body of evidence suggests that transmission of HEV as a foodborne pathogen is frequently zoonotic [91,92,93,94]. Although the presence of HEV seems to be higher in Europe than in the Americas, a recent meta-analysis suggests that the continents have nearly equal HEV seroprevalence [95]. Although this emerging foodborne virus is gaining ground, means of controlling it by inactivation remain scarce. The knowledge acquired in the quest to control HAV might shed light on the vulnerabilities of this new pathogen.

### 3.4. Laboratory Surrogates Used to Study Foodborne Viruses

The study of human norovirus genotypes has been slowed by limited access to (1) HuNoV-positive samples (feces and vomitus), (2) adequate biosafety containment (level 2), and (3) cell culture techniques that work on more than a few HuNoV genotypes. These obstacles have been overcome somewhat by using a variety of proxy viruses. Among these are murine norovirus 1 (MNV-1), Tulane virus (TV), feline calicivirus (FCV) and a few bacteriophages (e.g., MS2 and φX174) [96]. Some of these surrogates are phylogenetically closely related to HuNoV. For example, MNV-1, a genogroup V norovirus that only infects mice, has been found to be suitable as a surrogate in heat inactivation studies [97]. These substitutes exhibit key characteristics of HuNoV, such as persisting under conditions of extreme pH, humidity, and temperature for several days or months [98,99,100]. However, in terms of affinity, adhesion, and attachment, only TV has been shown to share several binding receptors with HuNoV [101,102,103], making it preferred in foodborne virus inactivation studies. The choice of a proxy virus must be reasoned, and caution must be exercised when extrapolating results directly to HuNoV. The development of an easy and accessible culture tools allowing the study of all HuNoV genotypes would definitely be ground-breaking.

### 3.5. Other Important Foodborne Viruses

Several other viruses have been implicated in outbreaks of illness involving the same food matrices as for HuNoV and hepatitis. These include rotaviruses, adenoviruses, astroviruses, parvoviruses, polioviruses, influenza, and other common cold or flu viruses [104,105]. The Aichi virus is also emerging as an important foodborne virus in several parts of the world [106,107]. Indirect contamination of food by viruses such as herpes simplex virus 1 (HSV-1) cannot be ruled out [108]. While these viruses are much less prevalent than HuNoV or the hepatitis viruses, viral control strategies should be adapted to inactivate them as well. In the current repertoire of viral inactivation methods, treatment with UV has been proving its effectiveness for a long time.

## 4. Mechanism of Viral Inactivation by UV, and Genome Repair Mechanisms

### 4.1. Generalities Regarding Inactivation by UV

UV has been used widely for more than a century to suppress microbial proliferation [109] in biological safety cabinets in research and medical laboratories [110], in water treatment facilities [111], hospitals [112], ambient bioaerosols [113], processing equipment in food industries [114,115], and in many other situations. Furthermore, continuous UV treatment as simple as the sunlight [116] or more complex designs, such as pulsed light machines [117] are amongst the vast possibilities of developed light sources [118,119,120,121,122] to control microorganisms. Although its effectiveness was discovered long ago, little has been added to its basic principle in our ongoing fights against pandemics [123] other than new designs such as UV-blasting robots [124]. The vast work in the field of UV disinfection ultimately led to the discovery of its antimicrobial mechanism of action, a key step toward viral control.

### 4.2. Nucleic Acid

Studies of the mode of action by which UV suppresses microbial proliferation have focused mainly on bacterial growth [125]. Briefly, UV photons have enough energy to break and form chemical bonds, causing, for example, dimer formation between consecutive bases in DNA or RNA [126]. Uracil, cytosine, thymine, and adenine may react, and the resulting damage may interfere with key functions such as genomic transcription and replication, leading ultimately to cell death [126]. Fortunately, most of the lethal mechanisms described for bacteria also affect viruses.

It is known that the genetic material within the viral capsid is a strong absorber of UV radiation [116], especially close to 254 nm, while other viral components such as proteins are minor absorbers [127]. UV is effective against both RNA viruses and DNA viruses, and sensitivity to UV is correlated strongly with genome length [116]. Pyrimidine dimers and particularly thymine dimers are the principal lethal photoproducts [128], and DNA viruses are, therefore, more sensitive than RNA viruses [127,129]. This is an important point in the food context, since the most problematic viruses have RNA genomes. Additionally, single-stranded DNA or RNA is more sensitive than double-stranded [130].

It has been shown with mengovirus that in addition to forming DNA or RNA dimers, UV can also destabilize and degrade the capsid and form covalent bonds between proteins and RNA [131], thus rendering the virus non-infectious [132]. This is important because the mechanism of action of UV seems to depend somewhat on the wavelength used. At 254 nm, the genome is targeted [133], whereas lower wavelengths (207–222 nm) appear to damage surface proteins that are essential for binding to host cells [123,134]. UV-C at 254 nm has been found not to damage the SARS-CoV-2 spike protein, nucleocapsid protein or virion morphology, while damaging the genome [135]. Furthermore, one study has also shown a significant loss of spike protein function following exposure to UV-A at about 343 nm [136]. This apparent relationship between exposure wavelength and potential for damaging viral proteins may be relevant to disinfection strategy.

### 4.3. Protein Damage

Although protein damage by UV plays a minor role in viral inactivation, no doubt remains about its occurrence [137,138]. Electron microscopy reveals structural modifications of major and minor proteins of the adenovirus capsid after low-intensity and medium-intensity UV treatments [137,139]. A similar effect has been observed after pulsed light treatment of MNV-1 [140]. These results all confirm UV as an important tool for controlling foodborne viruses based on damaging genetic material and key proteins. Another key component of viral inactivation by UV is the ability or inability of virions to repair damage inflicted on their genome.

### 4.4. Host Repair Mechanisms

While host repair mechanisms are beyond the scope of this review, it should be noted that photolyase can bind dimers, absorb energy, and use electron transfer to split dimers into their original DNA form [141]. The only virus known to possess such an enzyme is the fowl pox virus, a poultry pathogen [142]. UV, therefore, remains an effective antiviral disinfectant in food settings (surfaces and/or produces), notwithstanding the possibility of viral genome restoration by host photolyase.

## 5. Impact of UV Treatment on Foods and Food-Related Matrices (Liquids and Surfaces)

### 5.1. Non-Food Liquids

Water is relatively easy to disinfect with UV since it does not absorb light and can be mixed to ensure equal exposure of suspended particles including viruses. Transparent liquids such as phosphate-buffered saline, of which the absorbance in the 200–1100 range is close to zero, are therefore preferred for in vitro tests [143]. The amount of information available on the use of UV light to disinfect water is considerable. Indeed, phage MS2 is used for UV reactor validation in North America [133]. A classic review article on this subject was published in 2006 by Hijnen et al. [144] and comprehensive kinetic data were published in 2009 by Kowalski [111].

Pulsed light is a more recent technology and, therefore, less is known about its effectiveness for water disinfection. The first published account of its use for inactivation of viruses, including some foodborne, appears to be that of Roberts and Hope (2003), who obtained at least 4.8 log reductions in HSV-1, HAV, poliovirus 1, canine parvovirus, bovine parvovirus and non-foodborne viruses (Sindbis, vaccinia, encephalomyocarditis and Simian virus 40) with only two pulses of 1 J/cm^2^ each for less than 1 s of treatment time [117]. Lamont et al. (2007) later obtained 4 log reductions in poliovirus 1a and Group D adenovirus infectiousness in PBS, Jean et al. (2011) at least 4 log reductions in MNV-1 and HAV in PBS and Huang et al. (2017) at least 5 log reductions in MNV-1 and TV in PBS [143,145,146]. Inactivation was significantly less in PBS containing dissolved protein [117,143], which absorbs in the UV range. Table 1 summarizes the different viral inactivation results obtained using pulsed light (Table 1).

### 5.2. Liquid Foods

The inactivation of viruses in liquid foods has been studied using coconut water [150,151] and skim milk [152,153] challenged with phages MS2 and T1UV in two types of experimental set-up: collimated beam in a stirred batch reactor and a Dean vortex continuous reactor. Reductions in viral titer reached 4 log cycles for MS2 and more than 5 log for T1UV in coconut water [150,151] and at least 5 log for both MS2 and T1UV in skim milk [152,153]. It is widely known that the efficacy of UV treatments of liquid foods decreases with turbidity and suspended solids, which absorb or scatter the light before reaching the viral particles. However, this limitation can be overcome by reactor designs that promote turbulent flow to maximize the exposure of any virus present. In the above cases, UV transmittances as low as 9.7%/cm in coconut water and 0.57–0.89%/cm in skim milk were sufficient. The formation of potentially toxic compounds due to the action of UV light on food components was also examined. No cytotoxicity was observed when skim milk was treated with up to 0.17 J/cm^2^ [153] or coconut water with up to 0.4 J/cm^2^ [151], fluences that were high enough to reduce viral titers substantially.

### 5.3. Meat Products

The use of UV light as a single method for viral inactivation in meat products is possible, although results are modest. This is likely because the irregular surface shields viral particles from exposure and because meat proteins can absorb part of the illumination [154]. For example, only 1.23 and 1.17 log reductions in MNV-1 and HAV, respectively, were obtained on fresh chicken breast. The inactivation curve in both cases progressed without tailing, suggesting that higher fluences (up to 3.6 J/cm^2^) could increase inactivation. However, the sensory quality of the chicken meat was decreased at fluences above 1.2 J/cm^2^, at which only 0.5 log reductions were observed for both viruses. UV-C treatment combined with another decontamination technique would likely give better results [155]. A still modest but higher inactivation of FCV and *Escherichia coli* coliphages MS2 and φX174 (0.97–2.8 log reduction) was observed on pork liver, ham and sausage treated by pulsed light at fluences of 45–60 J/cm^2^ [148].

### 5.4. Fruits and Vegetables

Berries are delicate products very susceptible to damage and are usually just washed in tap water or chlorinated water during processing. These processes are insufficient to eliminate human pathogens that may be present. HAV and NoV have been implicated in numerous outbreaks related to fresh and frozen berries [116]. Decontamination of fresh and frozen strawberries, blueberries, and raspberries spiked with HAV and MNV-1 has been attempted (Table 2).

Even though UV-C was able to reduce viral titers on berry surfaces by up to 3 log cycles in some cases, its efficacy was limited and prolonging the treatment beyond 1.3 J/cm^2^ did not improve inactivation. Shielding by the irregular surface of berries is the likely cause of this, despite the UV reactor being designed with mirror-finish reflectors on all sides to maximize exposure. UV-C might nevertheless be effective on berries as part of a hurdle approach, since it has only a mild effect on fruit sensory quality and does not produce the toxic by-product furan [116]. The irregular surface of strawberries was a major obstacle for viral inactivation [157], which ranged from 1.9 to 2.6 log TCID5_0_/mL for FCV, HAV and Aichi virus, several log cycles lower than achieved on foods with smoother surfaces such as lettuce and green onions.

Pulsed light has been tested in a process by which berries are suspended in agitated water to avoid heating and facilitate berry movement and rotation and thereby allow more uniform exposure. Log reductions of 1.8 and 3.6 in MNV-1 titer were obtained for strawberry and raspberry, respectively [149], which were significantly higher than the inactivation obtained after immersion for an equivalent time (1 min) in water containing 10 ppm chlorine. As in the case of UV-C light [116], inactivation was greater on smoother-surfaced fruit (raspberry) than on strawberry. Furthermore, the higher efficacy of this water-assisted treatment versus chlorinated water suggests using this method as an alternative to chlorine, which can generate harmful by-products such as trihalomethanes.

Different research groups have tried to implement UV-C treatments to increase the safety of another product that is consumed fresh, namely lettuce. The efficacy of UV-C in this case is high, at least 4 log TCID_50_/mL for HAV, Aichi virus and FCV on Romaine lettuce [157] but not for phage MS2 on Iceberg lettuce [162]. In all cases, inactivation was initially rapid then tailed off. A low level of inactivation was also observed for MNV-1 [161] but with a log-linear decline.

Green onions may or may not be disinfected depending on the type of virus. High levels of inactivation (>5 log TCID_50_/mL) have been reported for HAV [156,157], but not for FCV, Aichi virus [157], human adenovirus type 41 and MNV-1 [156], in decreasing order of inactivation. Inactivation was negligible when viruses were internalized, which can occur during hydroponic growing by uptake through roots. These results are to be expected since UV-C is a superficial treatment.

### 5.5. Other Food Types

Viruses are very stable in low-moisture foods. UV-C light applied in combination with ozone and hydrogen peroxide vapor can inactivate practically 100% (4 log) of FCV and MNV in chocolate, but only 1 log in pistachios and <1 log in cornflakes [163]. Table 3 lists the inactivation of different viruses on foods other than vegetables by conventional UV-C light (Table 3). The efficacy of pulsed light has also been tested using MS2 virus on black pepper, garlic and chopped mint, but the titer was reduced by only 1.28 log in the best case [147].

### 5.6. Food Contact Surfaces

The virucidal efficacy of UV light on food contact surfaces has been tested using stainless steel and different types of plastics. Inactivation is theoretically favored on stainless steel because of the low degree of shielding, and was significant using UV-C, 4.4 log for MNV-1 and 2.6 log for HAV [159]. Both inactivation curves exhibited tailing, indicating that extending the exposure time would not increase inactivation proportionately. On the other hand, inactivation was completed when using pulsed light (5 log reductions) on stainless steel and PVC [143]. Using MNV-1 also, Vimont et al. (2015) observed reductions of 3.8 to 4.3 log on high-density polyethylene and stainless steel and total inactivation on PVC [140]. Both studies found that viral inactivation is decreased by several orders of magnitude when the surfaces are fouled.

## 6. Limitations of UV Treatment

Over the years, several limitations of UV as a sanitizing agent have been identified, such as its low penetrating power, the shadowing effect, its reduced efficacy in the presence of extraneous organic matter and the multiplicity of reactivation. Its penetrating power is low on materials such as fruit skins [164] and water [165], allowing many microorganisms to go unexposed. The penetration depth on food surfaces ranges from 6 µm to 2.2 mm, making UV unreliable for disinfecting porous food matrices [164]. The shadowing effect of rough or convoluted surfaces can be overcome somewhat using rotating lamps to illuminate from all possible angles. Berries with cavities, bivalve shellfish, and pork liver are examples of problematic foods. The presence of extraneous organic matter also has been shown to reduce the effectiveness of photon energy treatments by presenting an additional obstacle to illumination of food surfaces that may harbor viruses [166,167]. When possible, delicate washing of the matrix would help by removing at least some of this organic matter. Lastly, UV treatment should be expected to induce alterations (mutations) of the viral genome. If several damaged virions were to invade the same host cell, genome reassortment could allow a productive infection to occur with generation of a new strain [168,169]. This is called multiplicity of reactivation and has been described as a survival mechanism for foodborne viruses [170,171].

## 7. Legislation

The use of UV light in foods is regulated by different legislations, and in some countries, the approach used differs. While the USA regulates the use of the technology as such, other countries consider UV-treated foods as novel foods and legislate case by case. In these regulations, the use of UV light is considered not only due to its antimicrobial efficacy but also for other intended uses, such as the photogeneration of vitamin D. Some countries also differentiate their regulations depending on the type of light source and if there is no intended use for virus inactivation.

In the USA, conventional light sources and pulsed light technology have their own regulations. The use of low-pressure mercury lamps for food treatment is limited under regulation 21CFR179.39, which differentiates its use for surface microorganism control in food and food products, sterilization of water used in food production and reducing human pathogens and other microorganisms in fruit juices [172]. In this case, the US Food and Drug Administration does not specify fluence values but concluded that this should be achieved for individual usage situations in a manner consistent with good manufacturing practice [173]. The application of pulsed light technology to foods is regulated by the 21CFR179.41 [174], setting restrictions such as a maximum fluence of 12 J/cm^2^. The use of UV light for treating baker’s yeast with the purpose of synthetizing vitamin D2 is regulated by the 21CFR172.381 [175].

In the European Union, UV-light-treated foods are considered novel foods. The intended use is for the generation of vitamin D and it is noteworthy that the wavelength ranges differ for each case: 200–800 nm for mushrooms, not specified for baker’s yeast, 240–315 nm for bread and 200–310 nm for milk [176].

Canada allows the use of UV light using the CiderSure 3500 Ultraviolet (UV) light unit for treating unpasteurized and unfermented apple juice and cider products, implementation of which is aimed to inactivate *Escherichia coli* O157:H7 [177]. In India, raw milk treated with a Sure-Pure UV system has the status of “Process Approval” [173].

## 8. Conclusions and Future Trends

Published results on viral inactivation by UV light and pulsed or otherwise are consistent with what would normally be expected. UV light is a highly efficient virucide (directly altering the genetic material of viruses and modifying the composition of viral proteins) for transparent liquids and even opaque liquids if turbulent flow is maintained. On solid foods, surface irregularity limits inactivation. The limited amount of information available and the high variability in the response of different types of viruses to UV light preclude reaching any definitive conclusions about the commercial applicability of this technology. UV-C is somewhat less effective on meat, berries, and dry herbs. However, it may be combined with other technologies as part of a hurdle approach to achieve better results. The agitated-water-assisted UV treatment appears to be more effective than just rinsing with chlorinated water for disinfecting berries and deserves further exploration. Inactivation kinetics are, in some cases, log-linear and in others logarithmic followed by tailing. In the former case, higher fluences might yield better results for some virus–food pairs. UV can also be useful for food–contact surface decontamination. Acquiring additional knowledge on the impact of UV light on foodborne viruses and their laboratory surrogates remains a key process in our advancement towards better understanding of food safety and improving the future security of food industries and their customers.

## Figures and Tables

**Table 1:** Inactivation of viruses by pulsed light.

Virus	Matrix	Fluence (J/cm^2^)	Log Inactivation	References
Adenovirus	PBS *	5.6	4.0	[145]
Bovine parvovirus	PBS	1.0	4.3	[117]
Canine parvovirus	PBS	1.0	>6.5	[117]
Encephalomyocarditis	PBS	1.0	>5.9	[117]
*Escherichia coli* phage MS2	Black pepper	9.4	0.64	[147]
	Garlic	18.8	0.40	
	Chopped mint	18.8	1.28	
	Glass beads	9.4	4.87	
	PBS	3.8	>8	
	Swine liver	60	1.6	[148]
	Ham	60	0.97	
	Sausage	60	1.3	
HAV	PBS		>5.7	[117]
	PBS	0.05	4.8	[143]
	Stainless steel	0.06	5	
	PVC	0.091	5	
HSV-1	PBS	1.0	>4.8	[117]
MNV-1	Strawberry	1.27	1.8	[149]
	Raspberries	1.27	3.6	
	PVC	2.07	3	[140]
	Blueberry	22.5	3.8	[146]
	Strawberry	22.5	0.9	
	PBS	2.47	5.8	
	PBS	0.06	5.0	[143]
	PVC		5	
	Stainless steel	0.06	5	
	PBS	2.07	3.3	[140]
	Alginate	0.69	3.6	
	Hard water	4.84	3.9	
	Turbid water	3.45	3	
	Stainless steel	8.98	2.6	
Phage φX174	Swine Liver	60	2	[148]
	Ham	60	1.6	
	Sausage	60	1.6	
Poliovirus	PBS	0.28	4.0	[145]
		1	>6.7	[117]
Simian virus 40	PBS	1.0	3.7	[117]
Sindbis	PBS	1.0	7.2	[117]
TV	PBS	4.94	6	[146]
Vaccinia	PBS	1.0	>5.1	[117]

* PBS: phosphate-buffered saline.

**Table 2:** Inactivation of viruses on fruits or vegetables by UV-C.

Virus	Substrate	Fluence (J/cm^2^) for 1 log Reduction	Fluence (J/cm^2^) for 5 log Reduction	References
Adenovirus type 41	Green onions		0.240 (3 log) *	[156]
Aichi virus	Romaine lettuce		0.240	[157]
	Green onions		0.240 (3.66 log) *	
	Strawberries	0.240		
FCV	Romaine lettuce		0.240	
	Green onions		0.240 (3.92 logs) *	
	Strawberries	0.240 (2.28 log) *		
HAV	Frozen strawberries	0.212		[116]
		0.13		[158]
	Frozen blueberries	0.212		[116]
	Fresh strawberries	0.212		
		0.240 (2.60 log) *		[157]
	Fresh blueberries	0.212		
	Fresh raspberries	0.212		[159]
	Romaine lettuce		0.240	[157]
	Green onions		0.240	
			0.240	[156]
MNV-1	Fresh blueberry	1.331		[116]
	Fresh blueberry	1.2 (Dry)	1.2 (Water-assisted)	[160]
	Green onions	0.24		[156]
	Lettuce	0.6		[161]
Phage MS2	Iceberg Lettuce	0.019		[162]

* In parentheses: the reduction reached when differing from 1 log or 5 log.

**Table 3:** Inactivation of viruses by UV-C on foods other than vegetables.

Virus	Substrate	Fluence (J/cm^2^) for 1 log Reduction	Fluence (J/cm^2^) for 5 log Reduction	References
FCV	Chocolate		3.8 (4 log) *	[163]
	Pistachios	3.8 (2 log) *		
	Cornflakes	3.8		
HAV	Fresh chicken breast Stainless steel surface Chocolate Pistachios Cornflakes	3.6 0.3 3.8 (2 logs) * 3.8 3.8		[155] [163]
MNV-1	Fresh chicken breast	3.6		[155]
	Stainless steel surface		0.3	
		1.8 (2.65 log) *		[161]
	Chocolate		3.8 (4 log) *	[163]
	Pistachios	3.8 (1.5 log) *		
	Cornflakes	3.8 (0.6 log) *		
Phage MS2	Skim milk		0.150	[152]
			0.168	[153]
	Coconut water	0.02	0.12(4.20 log) *	[151]
			0.1	[150]
Phage T1	Skim milk	0.0062		[152]
			0.027	[153]
	Coconut water	0.005	0.03 (4.73 log) *	[151]
			0.03	[150]

* In parentheses: the reduction reached when differing from 1 log or 5 log.

## Data Availability

Not applicable.
